# Notch1—WISP-1 axis determines the regulatory role of mesenchymal stem cell-derived stromal fibroblasts in melanoma metastasis

**DOI:** 10.18632/oncotarget.13021

**Published:** 2016-11-02

**Authors:** Hongwei Shao, Long Cai, Mecker Moller, Biju Issac, Leiming Zhang, Mark Owyong, Anna Elizabeth Moscowitz, Roberto Vazquez-Padron, Freddy Radtke, Zhao-Jun Liu

**Affiliations:** ^1^ Department of Surgery, University of Miami School of Medicine, Miami, USA; ^2^ Hangzhou Red-Cross Hospital, Zhejiang, China; ^3^ Sylvester Comprehensive Cancer Center, University of Miami, Miami, FL, USA; ^4^ Yantai University, School of Pharmacy, Shandong, China; ^5^ Ecole Polytechnique Fédérale de Lausanne, Swiss Institute for Experimental Cancer Research, Lausanne, Switzerland

**Keywords:** cancer-associate fibroblasts, mesenchymal stem cells, Notch1, WISP-1/CCN4, melanoma

## Abstract

Mesenchymal stem cells-derived fibroblasts (MSC-DF) constitute a significant portion of stromal fibroblasts in the tumor microenvironment (TME) and are key modulators of tumor progression. However, the molecular mechanisms that determine their tumor-regulatory function are poorly understood. Here, we uncover the Notch1 pathway as a molecular determinant that selectively controls the regulatory role of MSC-DF in melanoma metastasis. We demonstrate that the Notch1 pathway's activity is inversely correlated with the metastasis-regulating function of fibroblasts and can determine the metastasis-promoting or -suppressing phenotype of MSC-DF. When co-grafted with melanoma cells, MSC-DF^Notch1^^−/−^ selectively promote, while MSC-DF^N1IC+/+^ preferentially suppress melanoma metastasis, but not growth, in mouse models. Consistently, conditioned media (CM) from MSC-DF^Notch1^^−/−^ and MSC-DF^N1IC+/+^ oppositely, yet selectively regulates migration, but not growth of melanoma cells *in vitro*. Additionally, when co-cultured with metastatic melanoma cells *in vitro*, MSC-DF^Notch1^^−/−^ support, while MSC-DF^N1IC+/+^ inhibit melanoma cells in the formation of spheroids. These findings expand the repertoire of Notch1 signaling as a molecular switch in determining the tumor metastasis-regulating function of MSC-DF. We also identified Wnt-induced secreted protein-1 (WISP-1) as a key downstream secretory mediator of Notch1 signaling to execute the influential role of MSC-DF on melanoma metastasis. These findings reveal the Notch1—WISP-1 axis as a crucial molecular determinant in governing stromal regulation of melanoma metastasis; thus, establishing this axis as a potential therapeutic target for melanoma metastasis.

## INTRODUCTION

Fibroblasts participate in the constitution of reactive tumor stroma [1]. They reside within tumor tissues and in the vicinity of tumor masses, also referred to as cancer-associated fibroblasts (CAF). CAF co-evolve with tumor cells and are critically involved in regulating tumor progression by eliciting a variety of soluble factors, structural components of the extracellular matrix (ECM), ECM remodeling enzymes [2, 3] and exosomes [4]. Moreover, CAF can escort tumor cells disseminating from primary lesions to the metastatic niche and support tumor cell survival and re-growth in the parenchyma of foreign tissues [5]. CAF also take part in determining organ-specific metastases by preselecting a subset of tumor clones from heterogeneous tumor cell populations in the primary lesion; thus, fostering these selected clones to be primed for metastasis to a specific distant organ where its microenvironment is optimal for re-colonization of selected clones [6]. Their contribution to primary and secondary malignancies as well as taking part in drug resistance and tumor recurrence [7, 8] makes CAF potential therapeutic targets.

CAF consist of a heterogeneous population of cells and can be derived from multiple origins, including infiltrated local tissue fibroblasts, recruited bone marrow–derived MSC (BMD-MSC), and perhaps trans-differentiated epithelial and endothelial cells [9–11]. BMD-MSC are one of the critical and major sources of CAF [10, 12, 13]. Approximately 40% of the total CAF within engrafted pancreatic cancers [10] and 60% of CAF in engrafted ovarian and breast cancers originate from BMD-MSC [14]. Hence, MSC-DF may serve as an on-site or off-site target for cancer therapeutic interventions on the TME.

Despite extensive evidence supporting the important tumor-regulating role of CAF, how CAF accomplish this regulation remains unknown. We have recently demonstrated a crucial role for Notch1 signaling in governing the tumor-regulating function of CAF. Using novel mouse models, in which the genetic activation or inactivation of Notch1 signaling specifically occurs in natural host stromal fibroblasts, we showed that CAF carrying elevated Notch1 activity significantly inhibited melanoma growth and invasion, while those with a null Notch1 promoted melanoma invasion [15]. Consistently, co-grafted experimental stromal fibroblasts carrying high Notch1 activity inhibited melanoma growth and angiogenesis in our mouse model [16]. These observations revealed that Notch1 signaling serves as a molecular switch, inversely controlling the tumor-regulating function of CAF. However, the fibroblasts investigated in these studies were not derived or fully derived from MSC, leaving the underlying mechanisms poorly investigated. Here, we created MSC-DF and utilized gain-of-function (GOF) and loss-of-function (LOF) approaches to comprehensively decipher the roles of Notch1 signaling and its downstream mediator, WISP-1, in determining the melanoma-regulating function of MSC-DF.

## RESULTS

### Generation and characterization of MSC-DF

**Figure 1:**
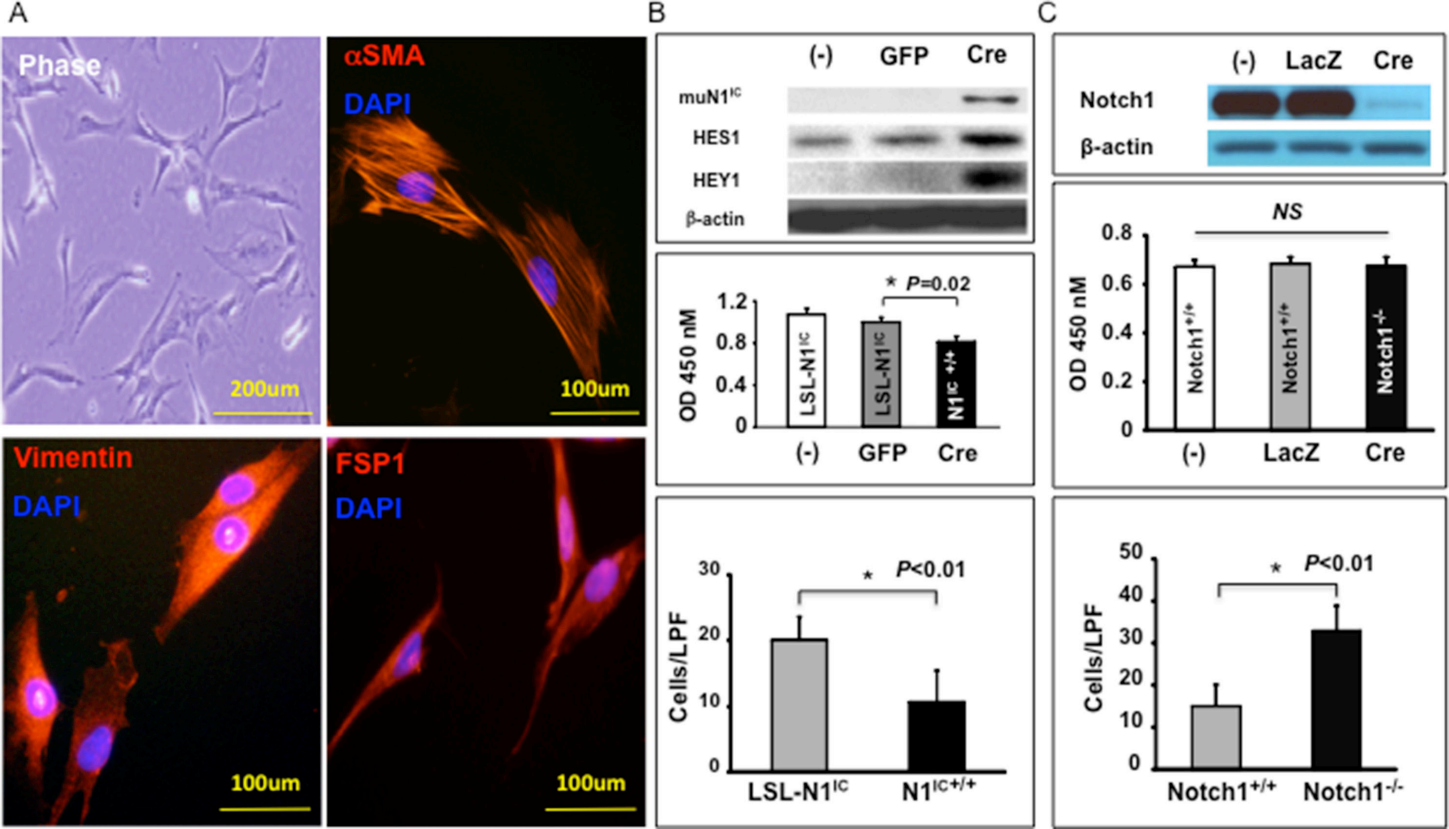
Generation and characterization of BM-derived MSC-DF (**A**) MSC-DF are αSMA^+^/vimentin^+^/FSP1^+^ by IF. (**B**) Effect of Notch1 activation on MSC-DF cell growth and migration. *top*: expression of mutant N1^IC^ and elevated levels of Hes1 and Hey1 protein in Cre/Lenti-transduced MSC-DF^N1IC+/+^. Decreased cell growth (*middle*) and migration (*bottom*) rates of MSC-DF^N1IC+/+^ vs. MSC-DF^LSL-N1IC^ (non-transduced (−) or GFP/Lenti-transduced). (**C**) Effect of Notch1 deletion on MSC-DF cell growth and migration. *top*: Notch1 deletion at protein levels; *middle*: comparable growth rate of MSC-DF^Notch1−/−^ vs. MSC-DF^Notch1+/+^; *bottom*: MSC-DF^Notch1−/−^ migrate faster than MSC-DF^Notch1+/+^ (LPF: low power field).

To explore the role of Notch1 signaling in determining the tumor regulatory function of MSC-DF, we first generated MSC-DF expressing N1IC. MSC-DF generated from ROSA^LSL-N1IC^ mice exhibited typical spindle-shaped fibroblast appearance and were characterized as α-SMA^+^, vimentin^+^ and FSP1^+^ cells through IF (Figure 1A). MSC-DF were then transduced with Cre-ires-GFP/Lentivirus to induce expression of N1IC resulting in enforced Notch1 activation. MSC-DF transduced with GFP/Lentivirus were used as a control. Lentivirus-transduced MSC-DF were sorted out by FACS (GFP+ cells). Expression of mutant N1IC (59Kda PEST-domain truncated form) in MSC-DF was validated by immunoblot (Figure 1B, top). Notch1 activation did not appear to impact on phenotypic stability of MSC-DF, since Cre-*ires*-GFP/Lentivirus-transduced cells were maintained as α-SMA^+^/vimentin^+^/FSP1^+^, so did GFP/Lentivirus-transduced cells (Supplementary Figure S1, left). MSC-DF^N1IC+/+^ exhibited slower growth and migration rates when compared to the MSC-DF^LSL-N1IC^ control (Figure 1B, middle and bottom), as tested by WST and transwell assays.

We also generated MSC-DF, which carry null Notch1. MSC-DF from *Notch1*^LoxP/LoxP^ mice were prepared and characterized (α-SMA^+^/vimentin^+^/FSP1^+^), then transduced with Cre-*ires*-GFP/Lentivirus (to delete *Notch1* gene) or GFP/Lentivirus (as control) identically as described above. Notch1 deletion was validated by immunoblot (Figure 1C, top). Similar to that observed in MSC-DF^N1IC+/+^, *Notch1* deletion did not impact the phenotypic stability of MSC-DF (Supplementary Figure S1, *right*). MSC-DF^Notch1−/−^ and control MSC-DF^Notch1+/+^ had comparable growth rates, while MSC-DF^Notch1−/−^ migrated faster than MSC-DF^Notch1+/+^ control (Figure 1C, middle and bottom). This indicated that loss of Notch1 did not affect cell proliferation, but enhanced cell migration of MSC-DF. These results demonstrated that Notch1 activity modulates biological functions of MSC-DF.

### Effect of MSC-DF^*N1IC+/+*^ and MSC-DF^*Notch1*−/−^ on melanoma cells *in vitro*

**Figure 2:**
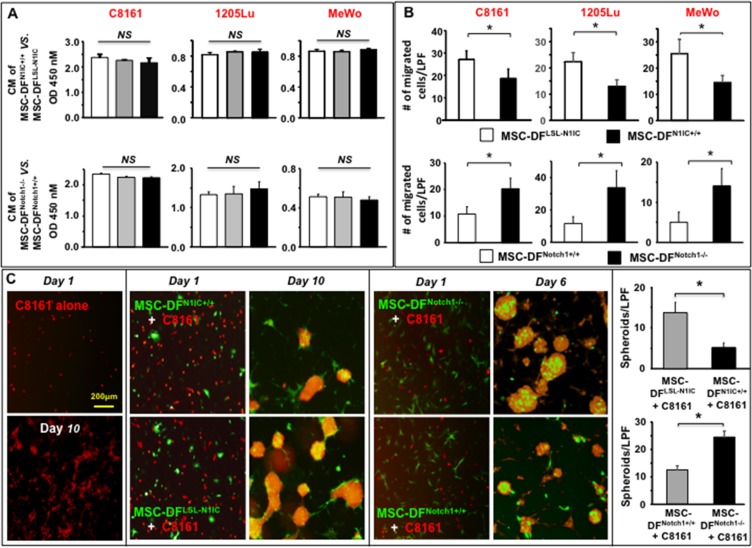
Differential effects of Notch1 activation and inactivation in MSC-DF on melanoma cell behavior *in vitro* (**A**) Cell growth of three melanoma cells is not affected by CM of MSC-DF^N1IC+/+^ or MSC-DF^Notch1−/−^. *NS*: no significance. White bars represent non-transduced cells and grey bars are GFP/lenti-transduced cells, while black bars are Cre/lenti-transduced cells. (**B**) Effect of CM from MSC-DF^N1IC+/+^ and MSC-DF^Notch1−/−^ on C8161 migration. CM of MSC-DF^N1IC+/+^ (*top*) inhibit while CM of MSC-DF^Notch1−/−^ (*bottom*) promote melanoma cell migration. *P < 0.01. (**C**) MSC-DF^N1IC+/+^ (GFP^+^) mitigate, yet MSC-DF^Notch1−/−^ (GFP^+^) increase spheroid formation of C8161 (DsRed^+^) in co-culture. C8161 alone don't form typical spheroid. Quantification of spheroids formed with MSC-DF^N1IC+/+^ or MSC-DF^Notch1−/−^ is showed in the left panel. Data are analyzed by Student's *t*-test and presented as mean ± SD based on three independent experiments. *P < 0.01.

We then investigated the role of MSC-DF^N1IC+/+^ and MSC-DF^Notch1−/−^ in regulating melanoma cell behavior *in vitro*. We first tested the effect of CM of MSC-DF^N1IC+/+^ vs. MSC-DF^LSL-N1IC^ and MSC-DF^Notch1−/−^ vs. MSC-DF^Notch1+/+^ on proliferation of 3 human metastatic melanoma cell lines (C8161, 1205Lu and MeWo). These three melanoma cells have different mutation backgrounds. 1205lu carries BRAF^V600E^ mutation. C8161 and MeWo cells don't have BRAF mutation, yet C8161 cells express high levels of CDK4/Kit. 5 × 10^3^ melanoma cells were cultured in CM overnight and cell proliferation was measured using the WST assay. No significant difference in melanoma cell growth was observed between cells treated with CM from MSC-DF^N1IC+/+^ vs. MSC-DF^LSL-N1IC^ and MSC-DF^Notch1−/−^ vs. MSC-DF^Notch1+/+^. Results of three melanoma cells are shown in Figure 2A.

Next, we examined the effect of CM on MSC-DF^N1IC+/+^ vs. MSC-DF^LSL-N1IC^ and MSC-DF^Notch1−/−^ vs. MSC-DF^Notch1+/+^ through transwell migration of three melanoma cells. Melanoma cells were seeded in the inserts, while the low chambers contained 50% CM. After 16 hours, the numbers of melanoma cells that passed through each well was counted. Compared to the CM of MSC-DF^LSL-N1IC^, CM of MSC-DF^N1IC+/+^ suppressed migration of melanoma cells (Figure 2B, *top*). In contrast, CM of MSC-DF^Notch1−/−^ considerably increased the migration of melanoma cells when compared to CM of MSC-DF^Notch1+/+^ (Figure 2B, *bottom*); thus, suggesting that Notch1 signaling may regulate MSC-DF to release soluble factor(s) that modulates the migration of melanoma cells.

Lastly, we conducted cell–cell (melanoma cells–MSC-DF) co-culture experiments to further examine the tumor-regulating effect of MSC-DF. MSC-DF^N1IC+/+^ vs. MSC-DF^LSL-N1IC^ and MSC-DF^Notch1−/−^ vs. MSC-DF^Notch1+/+^ (all MSC-DF were GFP^+^) were mixed with human C8161 metastatic melanoma cells, which were pre-transduced with DsRed/Lentivirus, respectively. Individually cultured C8161 metastatic melanoma cells grew more slowly than that co-cultured with MSC-DF. They did not form clusters until day 7. Some C8161 melanoma cells formed a few small clusters, but not typical spheroids (Figure 2C, *left*). When tumor cells were co-cultured with MSC-DF^N1IC+/+^ vs. MSC-DF^LSL-N1IC^, we observed that MSC-DF^LSL-N1IC^ induced C8161 to increase formation of typical spheroids, a characteristic of “cancer stem-like cell”. Spheroids were also formed faster (starting from day 4~5) in co-culture. However, MSC-DF^N1IC+/+^ exhibited a strong suppressive effect on spheroid formation by C8161 (Figure 2C, *middle*). In contrast, MSC-DF^Notch1−/−^ promoted melanoma cells to form larger and more spheroids than MSC-DF^Notch1+/+^ (Figure 2C). It is noted that the configurations of melanoma spheroids formed with MSC-DF^N1IC+/+^ and MSC-DF^LSL-N1IC^ vs. MSC-DF^Notch1−/−^ and MSC-DF^Notch1+/+^ appear somewhat different. MSC-DF^N1IC+/+^ and MSC-DF^LSL-N1IC^ were mainly located in the bottom and edge of spheroids, and tended to connect with other MSC-DF^N1IC+/+^ or MSC-DF^LSL-N1IC^ protruding from neighboring spheroids. However, MSC-DF^Notch1−/−^ and MSC-DF^Notch1+/+^ were mostly found on the top of spheroids and did not tend to connect with other MSC-DF^Notch1−/−^ or MSC-DF^Notch1+/+^ in neighboring spheroids. The reason for such a difference is unknown and left for future study. Similar results of the tumor-regulating effect of MSC-DF on spheroid formation by 1205Lu and MeWo melanoma cells were observed (Supplementary Figure S2).

Collectively, our results demonstrated that LOF of Notch1 results in MSC-DF promoting melanoma cell migration and spheroid formation, whereas GOF of Notch1 causes MSC-DF to suppress migration and spheroid formation of melanoma cells. Therefore, the data indicate that Notch1 signaling serves as a molecular switch that determines the tumor-regulating function of MSC-DF.

### MSC-DF^*Notch1*−/−^ selectively promote melanoma invasion and metastasis *in vivo*

**Figure 3:**
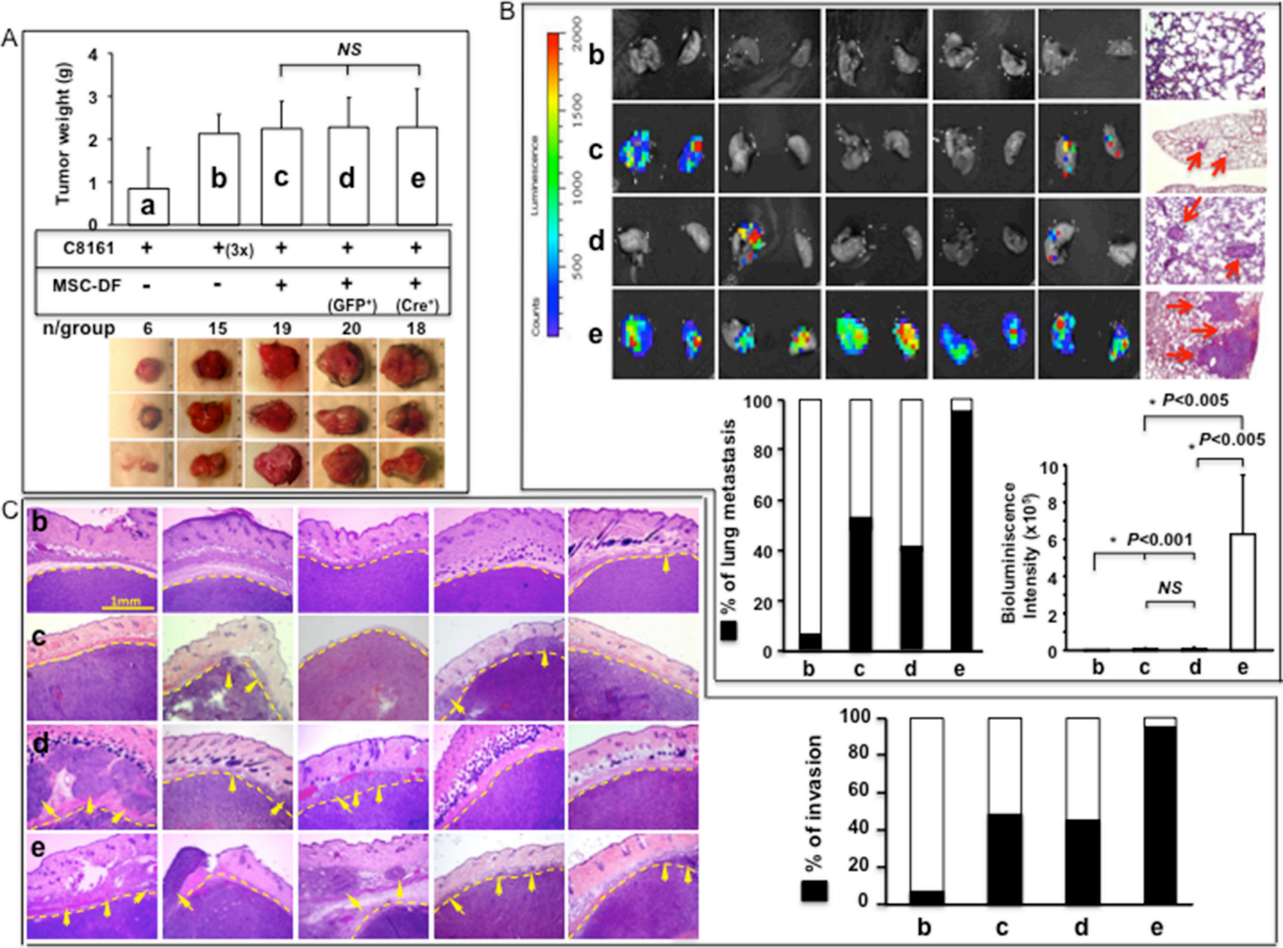
MSC-DF^Notch1−/−^ selectively promote melanoma invasion and metastasis *in vivo* (**A**) Melanoma growth in mouse skin. *top*: Tumor weight; *bottom*: three representative images of resected tumors/group. Solo-grafted melanoma grew slowly compared with co-grated melanoma (a). Growth rate of 3× solo-grafted melanoma was comparable to co-grafted melanoma cells (b). No significant difference for tumor growth with MSC-DF^Notch1−/−^ (e) *vs*. MSC-DF^Notch1+/+^ (non-transduced (c) or GFP/lenti-transduced (d)). (**B**) Tumor lung metastasis was robustly increased when co-grafted with MSC-DF^Notch1−/−^ *vs*. MSC-DF^Notch1+/+^. Five representative IVIS images of lungs/group are shown. Metastatic foci pointed by arrows in lung were detected by H&E staining. % of lung metastasis and tumor burden in lung are exhibited. (**C**) MSC-DF^Notch1−/−^ significantly promote melanoma Invasion. Five representative H&E images of tumor sections/groups are shown. Dash lines highlight tumor boundaries. Arrowheads point to melanoma cell invasion. % of local invasion detected by H&E staining is shown. In panel A, B, C: b: (3×) C8161; c: C8161 + MSC-DF^Notch1+/+^ (non-transduced); d: C8161 + MSC-DF^Notch1+/+^ (GFP/lenti-transduced); e: C8161 + MSC-DF^Notch1−/−^ (Cre/lenti-transduced).

We tested the effect of MSC-DF carrying null Notch1 on melanoma growth, invasion and metastasis using an identical co-graft mouse model (*n* = 8/group). Mice were sacrificed 6 weeks after co-grafting. Lung, heart, liver, spleen, brain, and kidney were harvested and scanned by IVIS to detect distant metastasis of skin melanoma. Melanoma cells were transduced with Luc2/Lentivirus. Melanoma growth on skin was comparable between MSC-DF^Notch1−/−^ vs. MSC-DF^Notch1+/+^ groups (Figure 3A). However, MSC-DF^Notch1−/−^ robustly increased lung metastasis, in terms of metastasis rate (~100%) and tumor loading in lung (bioluminescence signals), when compared to the MSC-DF^Notch1+/+^ group (Figure 3B). No distant metastasis was detectable in other major organs by IVIS. In our experimental condition, solo-xenografted C8161 (a) grew slowly and did not disseminate, even if 3× the amount of C8161 cells (b) were xenografted and tumor sizes were close to that of the co-grafted melanoma. When co-grafted with control MSC-DF (non-transduced MSC-DF (c) derived from *Notch1*LoxP/LoxP mice or MSC-DF^Notch1+/+^ (GFP-transduced (d)), melanoma could achieve approximately 40% of lung metastasis. When co-grafted with MSC-DF^Notch1−/−^ (e), a significant increase in melanoma local invasion was consistently observed when compared to that co-grafted with MSC-DF^Notch1+/+^ (Figure 3C). Overall, our data showed that turning ‘OFF’ Notch1 signaling in MSC-DF could increase melanoma invasion and metastasis, but not skin growth.

### *MSC-DF^N1IC+/+^* inhibit melanoma invasion and metastasis *in vivo*

**Figure 4:**
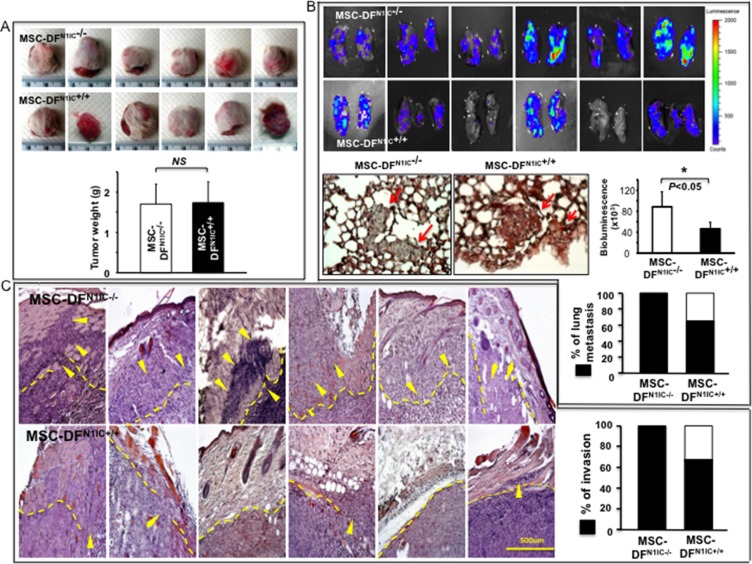
MSC-DF^N1IC+/+^ selectively inhibit melanoma invasion and metastasis *in vivo* (**A**) Growth of co-grafted melanoma in mice. *Top*: images and weights of tumors resected from each group (*n* = 6/group). No significant difference in growth of tumor co-grafted with MSC-DF^N1IC+/+^ vs. MSC-DF^LSL-N1IC^. (**B**) MSC-DF^N1IC+/+^ significantly diminished lung metastasis, both tumor loading in lung and % of metastasis. Metastatic foci pointed by arrowheads in lung sections were confirmed by H&E staining. (**C**) MSC-DF^N1IC+/+^ significantly inhibit tumor invasion. Six representative images of H&E staining of tumor tissues/groups are shown. Dash lines highlight tumor boundaries. Arrowheads point to invading tumor cells. Rate of local invasion in different groups is summarized.

To further study the tumor-regulating role of MSC-DF carrying high Notch1 activity in melanoma, cell mixtures of MSC-DF^N1IC+/+^ + Luc2^+^-C8161 vs. MSC-DF^LSL-N1IC^ + Luc2^+^-C8161 were co-grafted onto the skin of *SCID* mice (*n* = 6/group). Mice were sacrificed 6 weeks after co-grafting. Lung, heart, liver, spleen, brain, and kidney were harvested and scanned by IVIS to detect distant metastasis of skin melanoma. MSC-DF^N1IC+/+^ and MSC-DF^LSL-N1IC^ had a comparable effect on melanoma growth in skin (Figure 4A). However, MSC-DF^N1IC+/+^ suppressed melanoma lung metastasis significantly (Figure 4B). No metastasis in liver and other organs were detectable by IVIS. Decreased local invasion of melanoma, when co-grafted with MSC-DF^N1IC+/+^, was consistently observed when compared to that co-grafted with MSC-DF^LSL-N1IC^ (Figure 4C). The data revealed that melanoma invasion and metastasis, but not skin growth, could be inhibited by MSC-DF^N1IC+/+^. Our data demonstrated that turning ‘ON’ Notch1 signaling in MSC-DF could inhibit melanoma invasion and metastasis. To study the growth rate and fate of co-grafted MSC-DF, the ratio of MSC-DF/melanoma cells in the primary skin xenografts at the end of the experiments was examined by immunostaining. Melanoma cells are Luc2^+^ (stained with green fluorescent dye) and co-grafted MSC-DF are GFP^+^ (stained with red fluorescent dye). As shown in Supplementary Figure S3, the numbers of various MSC-DF in tumor tissue are very limited while melanoma cells are overwhelming. This suggests that the early period of time following co-grafting may be crucial in determining the final consequence of melanoma metastasis because there was relatively higher ratio of MSC-DF/melanoma cells so that MDC-DF could exert their metastasis-regulating effects more efficiently than that in the late stage. We quantified the amounts of various MSC-DF in each group. We found fewer MSC-DF^N1IC+/+^ compared to MSC-DF^LSL-N1IC^, whereas the numbers of MSC-DF^Notch1−/−^ and MSC-DF^Notch1+/+^ were comparable in tumor tissues. This is consistent with cell growth rates of MSC-DF^N1IC+/+^ vs. MSC-DF^LSL-N1IC^ and MSC-DF^Notch1−/−^ vs. MSC-DF^Notch1+/+^ *in vitro* as showed in Figure 1B and 1C (middle panels).

### WISP-1 is a downstream target of Notch1 signaling in MSC-DF

To elucidate the mechanism(s) for the Notch1-determined tumor-regulating role of MSC-DF, we conducted cDNA microarray analyses to identify the putative target genes of Notch1 signaling in MSC-DF. We examined and compared MSC-DF^Notch1−/−^ *vs.* MSC-DF^Notch1+/+^ because null *Notch1* in MSC-DF robustly enhances their metastasis-promoting function. Hence, Notch1 target gene(s) identified by this approach may serve as a potential therapeutic target for melanoma metastasis.

**Figure 5:**
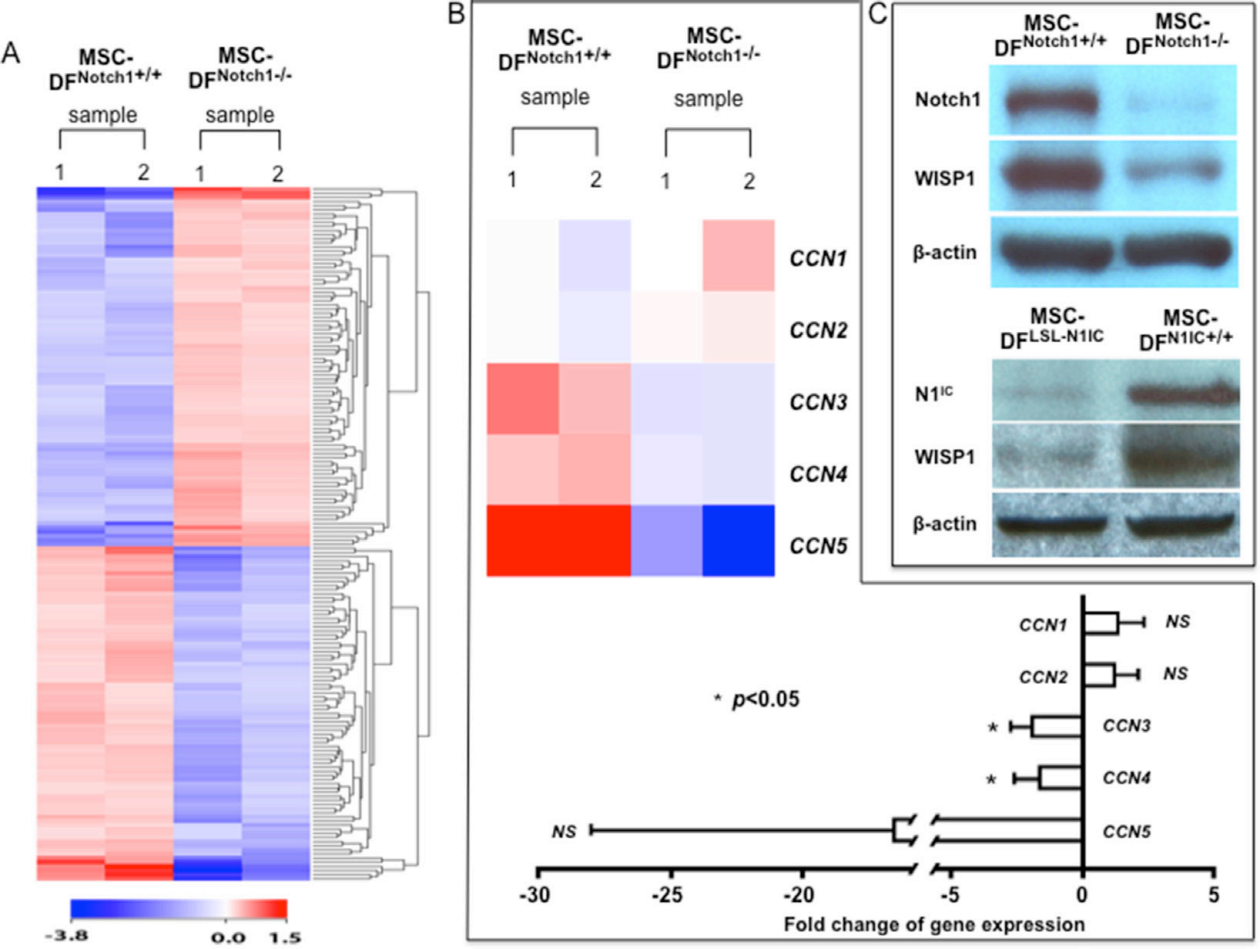
Expression of WISP1 depends upon Notch1 (**A**) Heatmap of differentially expressed gene profiles from duplicates of MSC-DF^Notch1−/−^ vs. MSC-DF^Notch1+/+^ by microarray analysis. (**B**) Heatmap of differentially expressed *CCN* family genes (*top*). Relative levels of *CCN1-5* genes in MSC-DF^Notch1−/−^ vs. MSC-DF^Notch1+/+^ (*bottom*), based on unpaired Student's *t*-test between the two conditions. (**C**) Deletion of Notch1 in MSC-DF^Notch1−/−^ (*top*) or expression of N1^IC^ in MSC-DF^N1IC+/+^ (*bottom*) and downregulation of WISP1 in MSC-DF^Notch1−/−^ are validated by immunoblotting.

The Illumina Mouse Whole-Genome-6 v2.0-based microarray analysis was performed. Gene expression profiles of MSC-DF^Notch1−/−^ *vs.* MSC-DF^Notch1+/+^ (control) were compared to discover differentially expressed genes (experiments in duplicates). 689 differentially expressed genes were found to be significant within *p*-value ≤ 0.05 and fold change ≥ 1.5, of which 417 were upregulated and 272 were downregulated in MSC-DF^Notch1−/−^. Clustering and visualization of 689 differentially expressed genes are shown in Figure 5A. The complete gene list is presented in Supplementary Table S1.

Our earlier findings about CM of MSC-DF^Notch1−/−^ in promoting melanoma cell migration suggested release of soluble molecule(s) by MSC-DF^Notch1−/−^. Therefore, we focused on soluble factor(s) produced by MSC-DF. We previously reported that constitutive activation of the Notch1 pathway resulted in an elevated expression of WISP-1/CCN4 in human fibroblasts [16, 17]. *WISP-1/CCN4* is indeed one of the 272 downregulated genes. Thus, we investigated gene expression profiling of the *CCN* family [18]. KEGG pathways were analyzed to derive genes of the *CCN* family. As summarized in Figure 5B, expression of *Nov*/*CCN3* and *WISP-1*/*CCN4* decreases 1.94-fold and 1.65-fold, respectively, in MSC-DF^Notch1−/−^, while changes of *Cyr61*/*CCN1, CTGF*/*CCN2 and WISP-2*/*CCN5* are not significant. Notch1 deletion and decreased WISP-1 in MSC-DF^Notch1−/−^ was confirmed by immunoblot (Figure 5C, *top*). Thus, microarray gene expression profiling indicated that *Nov*/*CCN3* and *WISP-1*/*CCN4* are downregulated upon *Notch1* deletion in MSC-DF. Consistently, expression of WISP-1 protein is elevated in MSC-DF^N1IC+/+^ (Figure 5C, *bottom*). These results revealed that WISP-1 is a downstream target of Notch1 signaling.

### WISP-1 mediates the Notch1-determined metastasis-regulating role of MSC-DF

**Figure 6:**
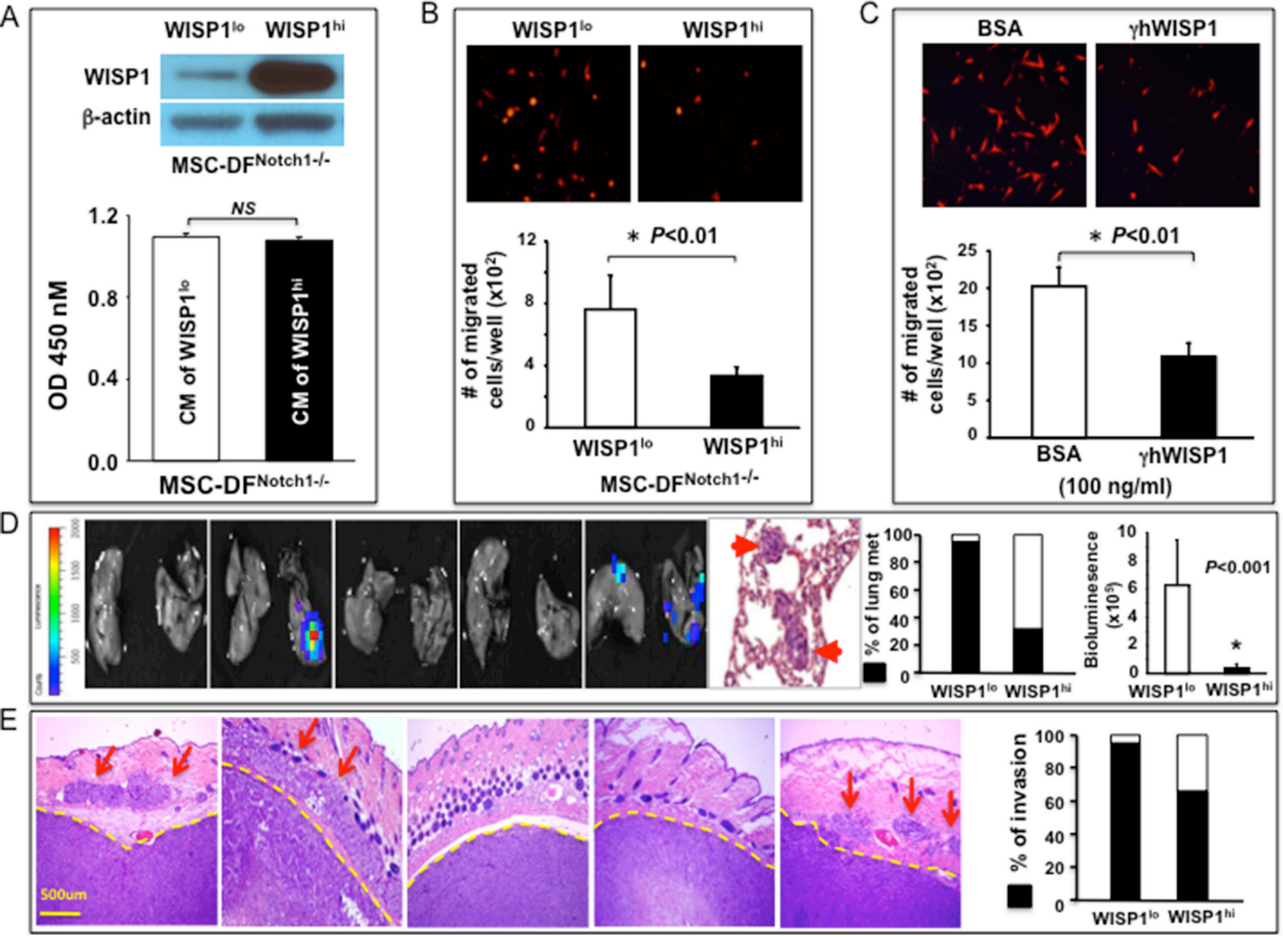
WISP-1 mediates Notch1-determined metastasis-regulating function of MSC-DF (**A**) *top*: Immunoblot shows reconstituted overexpression of WISP-1 in MSC-DF^Notch1−/−^ ; *bottom:* CM of WISP-1^hi^/MSC-DF^Notch1−/−^ and WISP-1^lo^/MSC-DF^Notch1−/−^ don't affect C8161 cell proliferation. (**B**) CM of WISP-1^hi^/MSC-DF^Notch1−/−^ inhibit DsRed^+^-C8161 migration. (**C**) Effect of γhWISP-1 on DsRed^+^ C8161 migration. Quantitative data in A, B, C are analyzed by Student's *t*-test and presented as mean ± SD of three independent experiments. (**D**) WISP-1^hi^/MSC-DF^Notch1−/−^ could largely reverse WISP-1^lo^/MSC-DF^Notch1−/−^-induced metastasis-promoting effects (see Figure 3B-e). Five representative IVIS images of lungs are shown. Metastatic foci in lung are confirmed by H&E staining. (**E**) WISP-1^hi^/MSC-DF^Notch1−/−^ significantly mitigate WISP-1^lo^/MSC-DF^Notch1−/−^-induced melanoma invasion (see Figure 3C-e). Five representative H&E images are shown. Arrows points to invading melanoma cells in skin tissues. Rate of local invasion is summarized.

To explore whether WISP-1 is functionally responsible for mediating the Notch1-determined regulatory function of MSC-DF, we reconstituted WISP-1 expression in MSC-DF^Notch1−/−^ by WISP-1/lentivirus transduction (Figure 6A, *top*). Transduced cells were named WISP-1^hi^/MSC-DF^Notch1−/−^ and compared with MSC-DF^Notch1−/−^ (WISP-1^lo^, which is E in Figure 3). First, we examined the effects of CM of WISP-1^hi^/MSC-DF^Notch1−/−^ vs. WISP-1^lo^/MSC-DF^Notch1−/−^ on proliferation and migration of melanoma cells. Although cell growth rates of melanoma cells cultured with CM of WISP-1^hi^/MSC-DF^Notch1−/−^ and WISP-1^lo^/MSC-DF^Notch1−/−^ were comparable (Figure 6A), CM of WISP-1^hi^/MSC-DF^Notch1−/−^ considerably inhibited melanoma cell migration (Figure 6B). Recombinant human WISP-1 (γhWISP-1) consistently inhibited C8161 cell migration (Figure 6C), demonstrating that WISP-1 suppresses melanoma cell migration. Consistent with previous reports [19], we observed that WISP-1 inhibited both the expression and phosphorylation of Rac1/Cdc42/RhoA in C8161 cells (Supplementary Figure S4).

Next, to test the effect of WISP-1^hi^/MSC-DF^Notch1−/−^ on melanoma growth and metastasis, we co-grafted cell mixtures of Luc2^+^-C8161 with WISP-1^hi^/MSC-DF^Notch1−/−^ vs. WISP-1^lo^/MSC-DF^Notch1−/−^ into *SCID* mice. There was no significant difference in melanoma skin growth between the two groups (data not shown). However, melanoma lung metastasis drastically decreased in the presence of WISP-1^hi^/MSC-DF^Notch1−/−^ in terms of both metastasis rate (down from ~100% to ~40%) and load (bioluminescent signals) in the lungs (Figure 6D, see Figure 3B-e for data of WISP-1^lo^/MSC-DF^Notch1−/−^ + Luc2^+^-C8161 group). Also, melanoma local invasion was inhibited when co-grafted with WISP-1^hi^/MSC-DF^Notch1−/−^ (Figure 6E, see Figure 3C-e for data of WISP-1^lo^/MSC-DF^Notch1−/−^ + Luc2^+^-C8161 group). These data revealed that reconstituted overexpression of WISP-1 could largely reverse the Notch1^−/−^-induced metastasis-promoting effect of MSC-DF; thus, demonstrating that the Notch1-determined melanoma metastasis-regulating role of MSC-DF is mediated primarily by WISP-1.

## DISCUSSION

Our study identified Notch1 signaling as a crucial molecular determinant in governing the tumor-regulating role of MSC-derived stromal fibroblasts and opens a new avenue to target the TME by either reprograming and converting CAF from ‘tumor promoters’ to ‘tumor suppressors’ through therapeutic activation of the Notch1 pathway or by directly exploiting Notch's downstream target, WISP-1. However, from the point of view of therapeutic practice, specifically activating the Notch pathway in CAF, while not simultaneously increasing the Notch signaling activity in tumor cells (herein melanoma cells) will be a key solution, since the biological function of Notch signaling is cell context-dependent [20, 21], and high Notch activity is oncogenic to a variety of tumors [22], including melanoma [23, 24]. Taking this into consideration and based upon the fact that a significant fraction of CAF in tumor tissue are derived from MSC, an alternative practicable strategy is to develop cell-based therapy through targeted delivery of therapeutic cells, i.e. autologous MSC-DF, pre-engineered ‘*ex vivo*’ to either overexpress WISP-1 or carry high Notch1 activity using numerous options, such as a gene therapy approach, a novel genome editing method, CRISPR/Cas9, to introduce *N1*IC, or applying the Notch-activating compound identified through a similar high-throughput screening method [25], into tumor tissue. Fibroblasts expressing high Notch activity tend to undergo cell cycle arrest [17, 26]. This characteristic makes MSC-DF carrying high Notch activity especially appealing as therapeutic cells because they will not expand uncontrollably after residing in the tumor tissue and are eventually cleared by immune cells. Therefore, they can be repeatedly administered to patients to enhance therapeutic efficacy.

This study reveals a selective role of the Notch1—WISP-1 axis in determining the regulatory role of MSC-DF in melanoma metastasis. However, it remains unclear whether such a metastasis-selective role is a unique trait of MSC-DF. In a previous study [16], we employed human dermal fibroblasts (FF2441), pre-transduced to overexpress N1IC, as experimental stromal fibroblasts to co-graft with human melanoma cells (1205Lu) on SCID mice. FF2441^N1IC^ inhibited melanoma cell skin growth. However, we were unable to investigate the effect of FF2441^N1IC^ on melanoma metastasis because grafted human 1205Lu melanoma cells did not metastasize in SCID mice. Also, in a different experimental mouse model, xenograftedmurine melanoma cells (B16) did not metastasize within the timeframe of our experiments in both GOF^Notch1^ and LOF^Notch1^ mice, in which the genetic activation or inactivation of Notch1 signaling specifically occurs in natural host stromal fibroblasts [15]. With respect to effects of different types of tumor stromal fibroblasts on melanoma growth, we observed that the loss of Notch1 in host stromal fibroblasts had little effect on B16 melanoma cell skin growth in LOF^Notch1^ mice. This result is consistent with the role of MSC-DF^Notch1−/−^ in regulating C8161 melanoma cell skin growth observed in the current study. However, B16 melanoma cell skin growth was retarded in GOF^Notch1^ mice, which is inconsistent with the role of MSC-DF^N1IC+/+^ in regulating C8161 melanoma cell skin growth revealed in the current study. Many factors may contribute to the varied responses of tumor growth in the genetic GOF^Notch1^ mouse model tested previously and in melanoma—MSC-DF^N1IC+/+^ cell co-graft mouse model applied in the current study. For example, different melanoma cells were studied in these varied models: murine B16 melanoma cells were tested in GOF^Notch1^ mouse models, while human C8161 melanoma cells were investigated in a co-graft model. A very low incidence of spontaneous metastasis of grafted B16 cells in the syngeneic murine melanoma model has been reported [27]. Typically resection of the primary tumor must take place in order for formation of distant metastases to occur. This is the reason why we used human metastatic C8161 melanoma cells in the current study, which was focused on exploring the role of Notch1 signaling in determining the function of MSC-DF in regulating melanoma metastasis. Besides, the nature of stromal fibroblasts investigated in these varied models are different. In the GOF^Notch1^ mouse model, endogenous natural fibroblasts were studied, whereas in the co-graft model, exogenously created MSC-DF were tested. In addition, the amount and location of stromal fibroblasts were different. There were fewer infiltrated/recruited CAF within the tumor mass, yet many CAF were located in the tumor capsule in the GOF^Notch1^ mouse model, whereas large numbers of MSC-DF were experimentally mixed and interwoven with melanoma cells in a co-graft model. Moreover, tumor—host interactions are different in these varied models. Interactions of murine melanoma cells and murine stroma were studied in GOF^Notch1^ mouse model, while interactions of human melanoma cells with murine stroma were examined in co-graft model. Furthermore, there were distinct immune statuses between mice used in the GOF^Notch1^ model and the co-graft model. The GOF^Notch1^ model uses immunocompetent mice while co-graft model utilizes immunodeficient mice. Future studies are warranted to elucidate the underlying mechanisms for the Notch1—WISP-1 axis-determined metastasis-selective role of MSC-DF.

Our finding of WISP-1 as a functional mediator of Notch signaling provides a practicable agent to control melanoma progression since a soluble molecule is easily administered. WISP-1 was initially identified as a Wnt1-responsive target [28]. We have demonstrated that the Notch1-induced WISP-1 expression is mediated by Wnt11, but not Wnt1, in fibroblasts [17]. In a previous study, we also observed that Notch1 activation-induced growth-inhibition of fibroblasts, which is partially mediated by WISP-1, was relievable when the Notch activation was countered with dominant-negative mutant of Master-mind like 1 (DN-MAML-1) [17]. It suggested that Notch1-dependent regulation of WISP1 is canonical RBP-JΚ dependent. However, future study is required to elucidate the precise mechanism for Notch1-dependent regulation of WISP1. While the role of WISP-1 is ill-defined and appears to be varied in different cancers, potential involvement of WISP-1 in several cancers has been reported [19, 28–30]. WISP-1 is a tumor-suppressor in lung cancer [30], but may correlate with colon cancer progression [28]. We previously tested the effects of knocking-down WISP-1 by shRNA in the presence of Notch1 signaling pathway activation on melanoma cell proliferation *in vitro* and melanoma skin growth *in vivo.* We showed that attenuation of Notch-upregulated WISP-1 expression in fibroblasts could significantly liberate the inhibitory effect of stromal fibroblasts, which is induced by high Notch1 activity, on melanoma growth [16]. In the current study, we did this the other way around and tested the effects of reconstituted overexpression of WISP-1 in MSC-DF^Notch1−/−^ on melanoma behavior *in vitro* and *in vivo.* In general, our results of both knocking-down and overexpressing WISP-1 in the presence of high or low Notch1 activity are consistent. Our data reveal that WISP-1 is a key downstream mediator of Notch1 signaling in executing the influential role of MSC-DF on melanoma progression. Taken together, our current and previous work demonstrates that WISP-1, released from stromal fibroblasts, is tumor-suppressive in melanoma. We have shown that WISP-1 is highly expressed adjacent to normal skin, both in the epidermis and dermal fibroblasts, but not within the melanoma lesion. Melanoma has undetectable levels of WISP-1 [16]. WISP-1 does not inhibit growth of melanoma cells [16], but strongly suppresses melanoma cell migration *in vitro*. This may explain why MSC-DF^Notch1−/−^ and MSC-DF^N1IC+/+^ primarily affect melanoma metastasis, while having little effect on melanoma skin growth in co-graft experiments. Our findings of WISP-1 in inhibiting melanoma metastasis are consistent with previous observations of lung cancer metastasis [30]. The mechanisms for WISP-1 in suppressing lung cancer cell motility and invasion were attributed to the inhibition of Rac activation [19]. We also observed that WISP-1 inhibited phosphorylation of RhoA/Rac1/CDC42 in C8161 melanoma cells. However, it is noteworthy that responses of different melanoma cells to WISP-1 vary *in vitro*. WISP-1 is faintly inhibitory to growth of WM278 and WM3899, but does not suppress Sbcl2 melanoma cells [16], similar to its effect on C8161. Furthermore, WISP-1 does not mitigate migration of 1205Lu melanoma cells [16], but significantly suppresses C8161 migration. The mechanism for varied responses of different melanoma cells to WISP-1 *in vitro* also requires further study.

It is a captivating observation that Notch1 activation or inactivation in MSC-DF can significantly yet oppositely regulate melanoma spheroid formation, a characteristic of cancer stem cells in which tumor-initiating cells and metastasis-initiating cells reside [31]. In response to MSC-DF carrying varied Notch1 signaling activities, the ability of melanoma cells to form spheroids in co-culture experiments *in vitro* is consistent with their capability to metastasize in co-graft experiments *in vivo*. In sum, our data reveal that Notch1 signaling activity is inversely correlated with the tumor-regulating function of MSC-DF and highlight Notch1 signaling as a molecular switch to control the tumor-regulating function of stromal fibroblasts in determining tumorigenesis and metastasis.

## MATERIALS AND METHODS

### Mice and skin co-graft models of melanoma

*Notch1*^Loxp/LoxP^ mice were described [32]. *ROSA*^LSL-N1IC^ (#006850) mice, which carry *STOP* codon floxed Notch1 intracellular domain (LSL-N1^IC^) allele knocked-in in *ROSA* mice, were purchased from The Jackson Lab (Bar Harbor, ME). *SCID* mice were purchased from Charles River (Wilmington, MA). Mice were maintained at the DVR animal facility under standard conditions. All animal studies were approved by the University of Miami Institutional Animal Care and Use Committee (IACUC). To perform co-grafting experiments, 2 × 10^6^ cell mixtures of melanoma cells and MSC-DF (at a ratio of 1:1) suspended in 0.1 ml of saline were injected (*intradermally*) into the dorsal skin of 8~10-week old male *SCID* mice.

### Cells, cell proliferation and migration assays

Murine MSC were enriched by culturing BM-mononuclear cells in MesenCult^®^ medium supplemented with *MSC Stimulatory Supplements* (#05502; StemCell Technologies, Vancouver, Canada) for 10-day with periodic medium changes. These MSC were characterized as CD73^+^/CD105^+^/Lin^−^. MSC were subsequently cultured with complete DMEM (Invitrogen, Carlsbad, CA) for an additional 2 weeks to differentiate into fibroblasts. Human metastatic melanoma cells (C8161 [33], 1205Lu [23] were cultured in W489 medium as described [23]. MeWo (ATCC^®^ HTB-65™) were cultured in DMEM with 10% FBS except for co-culture.

Cell growth was tested using the WST cell proliferation kit (BioVision, Mountain Views, CA) according to the manufacturer's instruction. To test the effect of CM, MSC-DF's supernatant cultured in serum-free DMEM for 2 days was applied to (100 μl/well) 5 × 10^3^ melanoma cells pre-plated on 96-well plates. The cells were cultured with or without CM overnight before the WST assay. Cell migration was tested using BD Falcon FluoroBlok™ Systems with 8μm porous membrane Insert (BD Biosciences, Rockville, MD). 5 × 10^3^ cells were suspended in 0.5 ml serum-free DMEM and seeded in inserts to migrate towards low chamber filled with 0.7 ml of 50% CM. After 16 hours, migrated cells (GFP^+^ or DsRed^+^) were counted under a fluorescence microscope. Both cell growth and migration assays were tested in triplicates and assays were repeated three times.

### Lentivirus and cell transduction

GFP/lentivirus was described [23]. Cre-*ires*-GFP/lentiviral, Luc2^+^/lentiviral, DsRed/lentiviral and murine WISP-1/lentiviral vectors were constructed by inserting individual cDNA into *pLenti6* (Invitrogen) vector using standard molecular cloning technique and confirmed by DNA sequencing. Production of pseudotyped lentivirus and transduction of cells were performed as described [23]. Transduced cells were cultured with a regular complete medium for 3 days and sorted by FACS then tested in subsequent analyses.

### Bioluminescence imaging of IVIS

D-luciferin was injected intra-peritoneally 10 minutes prior to imaging (150 mg/kg). Mice were anesthetized with isoflurane and the whole-body was scanned using IVIS 200B (PerkinElmer, Waltham, MA) with a 1 minute capture, medium binning. Following the whole-body scan, major organs were harvested and re-scanned. Scan was completed within 30 minutes from D-luciferin injection. Bioluminescence signal was quantified using the Living Image software and reported as total light emission within the region of interest (photon/s). A signal was defined as positive when it was greater than the sum of the mean background signal plus 2 standard deviations (SD) of the background signal.

### Histology, immunofluorescence (IF) and immunoblot

H&E was performed as described [16]. To do IF, cells were fixed on a glass plate with 2% paraformaldehyde for 10 minutes. Following a PBS wash, cells were blocked with Protein Block (Dako, Carpinteria, CA) then incubated with antibodies (Abs) against αSMA, Vimentin (ab18460, ab8978, Abcam), FSP1 (GTX89197, Genetex, Irvine, CA), and then with Invitrogen Alexa Fluor^®^ 594–anti-mouse IgG (A21203) or –anti-goat IgG (A211058), respectively. Nuclei were stained with DAPI (Sigma-Aldrich, St. Louis, MO). Immunoblot was performed as described [34]. Membranes were probed with Abs (Abcam) to two Notch1: ab52627 for Notch1 & ab8925 for mutant N1^IC^ (muN1^IC^, 59Kd), WISP1 (sc-25441, Santa Cruz Biotechnologies, Santa Cruz, CA), or β-actin (A1978, Sigma-Aldrich) accordingly. In all immunofluorescence staining experiments, isotype-matched non-specific Ab was used as control.

### Gene microarray, data analysis and statistics

Gene microarray expression data obtained by hybridization of total RNA to the Illumina MouseWG-6_V2_0_R3_11278593_A platform was loaded on GeneSpring™ 13.0 software from Agilent and subjected to rigorous quality control. All 45281 probes were subjected to an unpaired Student's *t*-test between the two conditions. Multiple testing corrections were not performed and significance of genes was determined using nominal *p*-values. Clustering of the 689 differentially expressed genes was performed using *Euclidean* distance based similarity measure and *Average* linkage rule. Gene microarray data has been submitted to GEO (accession number: GSE65316). For all other experiments, data were statistically analyzed using two-tail Student's *t*-test and are expressed as the mean ± SD. The values are considered statistically significant when *p* < 0.05.

## SUPPLEMENTARY MATERIALS




