# Supplementary material for: Notch1—WISP-1 axis determines the regulatory role of mesenchymal stem cell-derived stromal fibroblasts in melanoma metastasis
Source: Oncotarget. 2016 Nov 2;7(48):79262–73. doi: 10.18632/oncotarget.13021 (PMC5346712; doi:10.18632/oncotarget.13021)
Supplement: Supplementary file 1 [file oncotarget-07-79262-s001.pdf]

# Notch1—WISP-1 axis determines the regulatory role of mesenchymal stem cell-derived stromal fibroblasts in melanoma metastasis

## Supplementary Materials

**Supplementary Table S1: List of differentially expressed genes.** See Supplementary_Table_S1

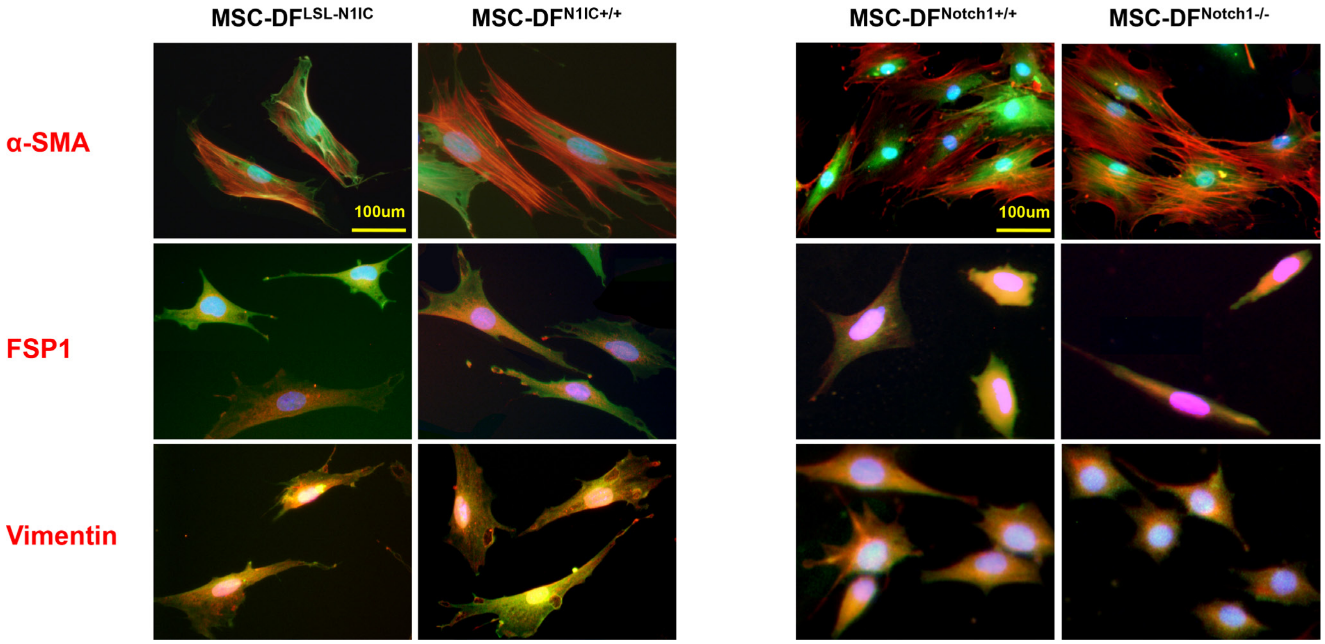


**Supplementary Figure S1: MSC-DF$^{N1IC+/+}$ and MSC-DF$^{Notch1-/-}$ are αSMA$^{+}$/vimentin$^{+}$/FSP1$^{+}$.** MSC-DF$^{N1IC+/+}$ vs. MSC-DF$^{LSL-N1IC}$ and MSC-DF$^{Notch1-/-}$ vs. MSC-DF$^{Notch1+/+}$ were stained by IF to detect cellular expression of αSMA/vimentin/ FSP1 as described in "MATERIALS AND METHODS".

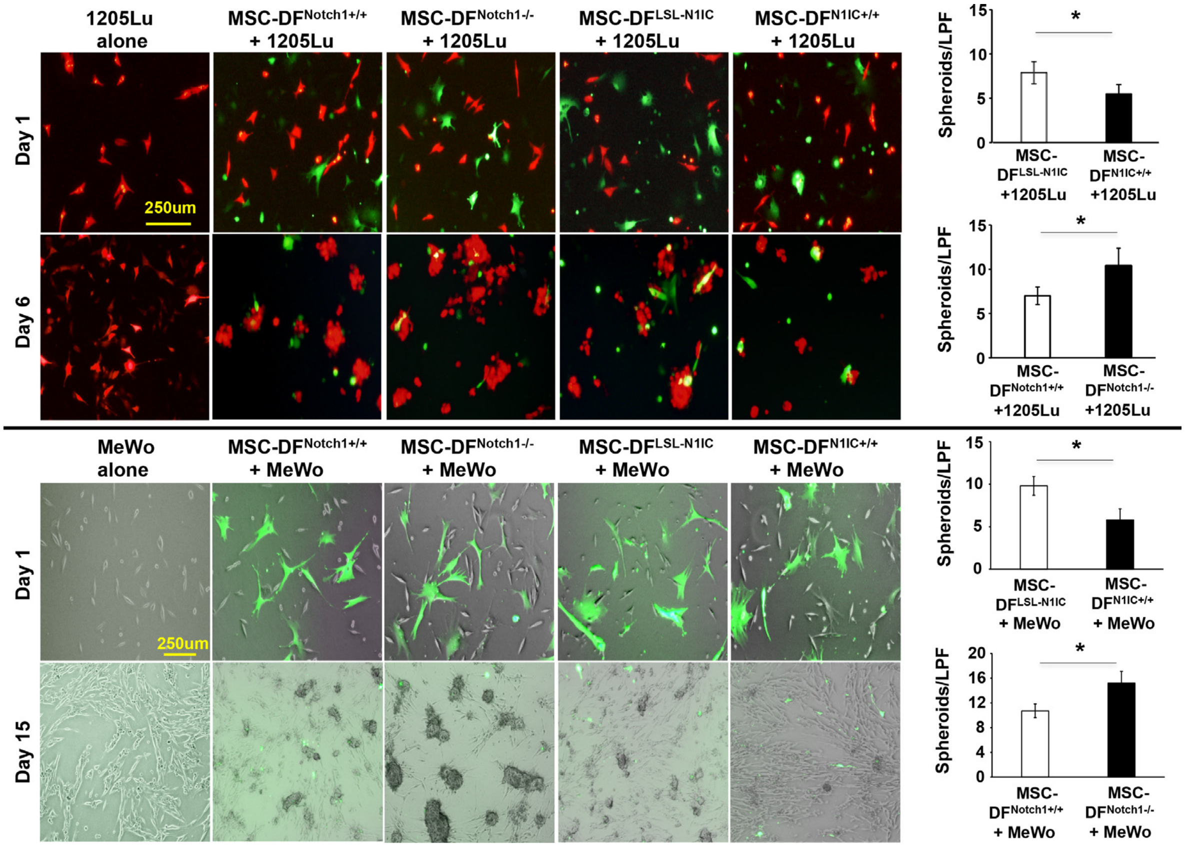


**Supplementary Figure S2: MSC-DF$^{N1IC+/+}$(GFP$^{+}$) mitigate, yet MSC-DF$^{Notch1–/–}$(GFP$^{+}$) increase spheroid formation of DsRed$^{+}$-1205Lu (*top*) and MeWo (*bottom*) in co-culture (Note: MeWo are not transduced with DsRed/Lentivirus and are DsRed$^{–}$).** Either DsRed$^{+}$-1205Lu or MeWo alone don't form typical spheroids. Cell co-culture was conducted as described in "MATERIALS AND METHODS", but the conditions suitable for the formation of spheroid by different melanoma cells vary slightly. For DsRed$^{+}$-1205Lu, ratio of DsRed$^{+}$-1205Lu : MSC-DF was 1:2 and cell mixtures were seeded into 6 well plates at a density of $2 \times 10^4$/well and cultured in serum$^{–}$/Ca$^{+}$/insulin$^{+}$ W489 and DMEM mixture (1:1). Spheroids formed in about 5 days. In the case of MeWo, ratio of MeWo : MSC-DF was 2:1 and cell mixtures were seeded into 6 well plates at a density of $2 \times 10^4$/well and cultured in serum$^{+}$/Ca$^{+}$/insulin$^{+}$ W489 and basal DMEM mixture (1:1). Spheroids formed in > 10 days. Quantification of spheroids formed with MSC-DF$^{N1IC+/+}$ or MSC-DF$^{Notch1–/–}$ is showed in the right panel. Data are analyzed by Student's *t*-test and presented as mean ± SD based on three independent experiments. *$P < 0.01$.

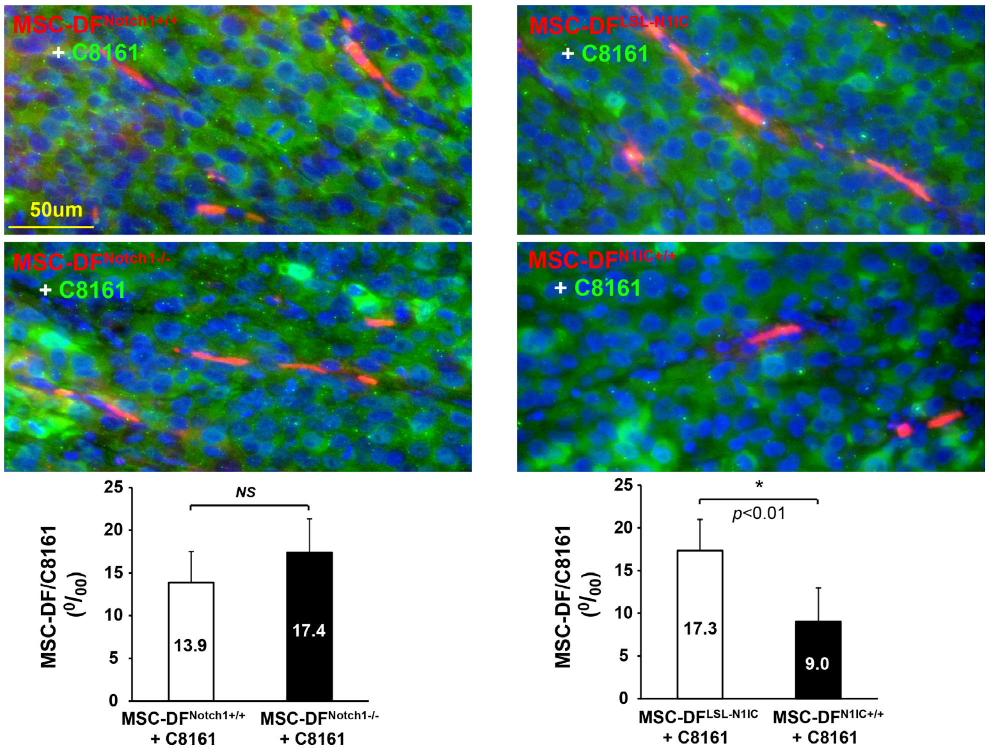


**Supplementary Figure S3: Amounts of various types of co-grafted MSC-DF in the primary skin xenografts at the end of the experiments.** Sections of skin tumor tissues resected at 6 weeks post co-grafting were subjected to immunostaining to detect amounts of co-grafted MSC-DF. Melanoma cells are Luc2$^+$ [stained with green fluorescent dye: the 1st Ab: anti-Luc (ab81823), the 2nd Ab: AlexaFluor®488-conjugated anti-IgG, Invitrogen A11055)] and co-grafted MSC-DF are GFP$^+$ [stained with red fluorescent dye: AlexaFlour®594-conjugated anti-GFP Ab (Thermoscientific A-21312)]. Amounts of MSC-DF in each group were quantified by examination of the ratio of MSC-DF/melanoma cells ($^0/_{00}$) per high power field (HPF). Representative images are shown. All data are mean ± SD based on counts of cells from HPF of 5 random sections per tumor tissue ($n$ = 8 tumors/group).

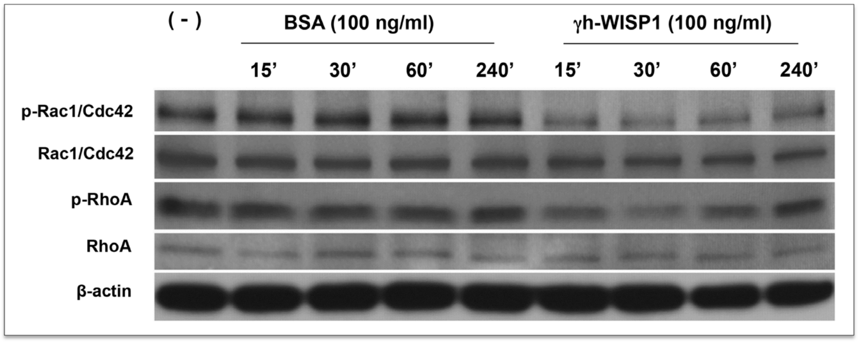


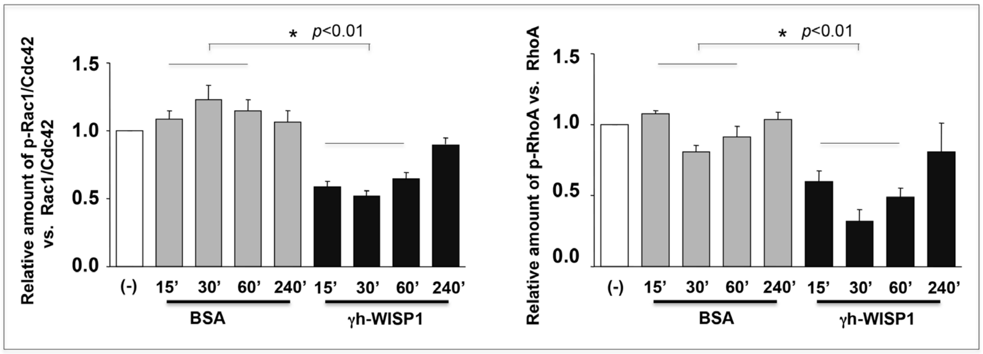


**Supplementary Figure S4: Phosphorylation of RhoA/Rac1/CDC42 is inhibited in C8161 melanoma cells treated with γhWISP1.** *top*: representative images of immunoblots; *bottom*: quantification of phosphorylation of Rac1/CDC42 and RhoA upon γhWISP1 treatment is based on three independent experiments. Relative levels of proteins are normalized by β–actin levels. Relative amounts of phosphorylated Rac1/CDC42 and RhoA versus total Rac1/CDC42 and RhoA are compared with that in untreated cells which is set at “1”.
