# Supplementary material for: Notch1—WISP-1 axis determines the regulatory role of mesenchymal stem cell-derived stromal fibroblasts in melanoma metastasis
Source: Oncotarget. 2016 Nov 2;7(48):79262–73. doi: 10.18632/oncotarget.13021 (PMC5346712; doi:10.18632/oncotarget.13021)
Supplement: Supplementary file 2 [file oncotarget-07-79262-s002.pdf]

Symbol	p value	Fold Change
Pcolce2	0.0022	12.022
S100a7a	0.0175	11.384
Pcolce2	0.0042	8.252
1700012B09Rik	0.0279	4.944
D1Ertd471e	0.0022	4.666
Serpinb2	0.0103	4.528
Cbln1	0.0016	4.203
Adra2a	0.0223	4.014
Lce1f	0.0463	3.891
Nes	0.0195	3.327
Nme7	0.0464	3.166
LOC381716	0.0099	3.094
Slco4a1	0.0010	3.028
Nme7	0.0268	2.980
Scx	0.0026	2.980
Tnc	0.0196	2.942
Elovl4	0.0269	2.916
Rbbp4	0.0331	2.814
Itga3	0.0337	2.773
Lce1g	0.0199	2.715
Rbm38	0.0334	2.694
Srpx2	0.0400	2.685
Srm	0.0373	2.681
Unc5c	0.0092	2.657
2010204K13Rik	0.0063	2.596
Gnb4	0.0496	2.591
Ybx1	0.0429	2.580
Cxadr	0.0297	2.577
Rcan1	0.0065	2.562
Unc5c	0.0094	2.543
Mboat1	0.0124	2.533
Klf5	0.0425	2.521

Symbol	p value	Fold Change
Cdkn1a	0.0468	-15.447
Dcn	0.0307	-14.538
Clec11a	0.0278	-10.878
Ifi27	0.0396	-9.163
Dcn	0.0303	-9.007
Ifitm1	0.0105	-5.813
Serpina3h	0.0411	-4.979
Igf1	0.0456	-4.224
Nfatc4	0.0235	-4.171
Acss2	0.0357	-3.518
Fam13c	0.0350	-3.479
Cxcl12	0.0317	-3.224
Cd248	0.0309	-3.218
Clip4	0.0320	-3.042
Gsdmdc1	0.0216	-3.036
Dnm3os	0.0481	-3.002
Psmb8	0.0439	-2.989
Tens1	0.0002	-2.850
1200015N20Rik	0.0309	-2.849
Tens1	0.0036	-2.823
4933439C20Rik	0.0196	-2.816
Gpnmb	0.0426	-2.802
Rassf2	0.0314	-2.769
Blvrb	0.0201	-2.656
H2-Q5	0.0212	-2.648
Uap1l1	0.0239	-2.648
LOC100046802	0.0050	-2.616
Tle6	0.0309	-2.602
Lama1	0.0453	-2.568
Gsdmdc1	0.0130	-2.567
Ube1l	0.0235	-2.565
Mgst1	0.0180	-2.557

Frap1	0.0421	2.452	Commd3	0.0170	-2.523
Mtif3	0.0293	2.449	2900062L11Rik	0.0403	-2.512
Kif5c	0.0379	2.442	Apobec1	0.0197	-2.500
LOC100044702	0.0482	2.436	Idh1	0.0432	-2.489
Exosc6	0.0377	2.374	4930533K18Rik	0.0327	-2.486
Cxadr	0.0374	2.371	Hcn2	0.0188	-2.480
Cfl1	0.0073	2.343	LOC621823	0.0358	-2.473
Mtvr2	0.0084	2.326	Rnf135	0.0296	-2.462
Limk1	0.0123	2.318	Larp6	0.0212	-2.443
Syn1	0.0054	2.318	9030024J15Rik	0.0290	-2.420
Sdcbp2	0.0141	2.318	Nipsnap1	0.0233	-2.413
Hak-pending	0.0041	2.301	Lox	0.0393	-2.402
5031439G07Rik	0.0279	2.252	C130092E12	0.0495	-2.382
Rad23a	0.0026	2.252	Zfp521	0.0072	-2.380
2900060B14Rik	0.0479	2.244	EG630499	0.0244	-2.358
Ddx20	0.0473	2.244	H2-D1	0.0089	-2.356
Mapk6	0.0147	2.242	LOC98434	0.0057	-2.304
D030034I04Rik	0.0080	2.222	Rdm1	0.0482	-2.300
Copg	0.0091	2.212	Grem1	0.0347	-2.300
5832418A03	0.0188	2.210	Htatip2	0.0228	-2.296
Ripk2	0.0353	2.201	Dtx3l	0.0230	-2.268
Tnc	0.0134	2.200	Asb2	0.0474	-2.234
Ptprk	0.0190	2.198	H2-T23	0.0150	-2.233
EG665685	0.0345	2.187	Blvrb	0.0411	-2.226
Copg	0.0125	2.170	Bax	0.0416	-2.211
Mtvr2	0.0210	2.169	Eno2	0.0214	-2.208
Jam2	0.0343	2.168	Pex13	0.0401	-2.185
Spink2	0.0309	2.161	Ebp	0.0165	-2.184
Nol1	0.0374	2.158	Pon3	0.0360	-2.171
Kti12	0.0222	2.125	Ddit3	0.0013	-2.164
Ibrdc3	0.0073	2.115	Ephx2	0.0462	-2.162
Ccl25	0.0081	2.099	Ilvbl	0.0143	-2.146
Fxyd5	0.0293	2.097	Parp14	0.0285	-2.144
Gclm	0.0397	2.091	Irf1	0.0299	-2.142

Cirh1a	0.0054	2.083	Cd248	0.0418	-2.139
Ddx19a	0.0077	2.081	Sfrp1	0.0441	-2.135
Ppbp	0.0049	2.081	Ogt	0.0491	-2.123
Mtf1	0.0099	2.072	Bst2	0.0303	-2.112
Syn1	0.0078	2.072	C2	0.0466	-2.104
Garnl4	0.0332	2.071	1190002H23Rik	0.0463	-2.099
Fscn1	0.0183	2.060	Ppnr	0.0407	-2.078
Atp5k	0.0028	2.056	Mkx	0.0102	-2.068
Marcksl1	0.0082	2.055	Arsa	0.0395	-2.052
Gm567	0.0031	2.053	Nrbp2	0.0393	-2.051
Ccrn4l	0.0312	2.050	LOC236604	0.0277	-2.036
Rangrf	0.0068	2.036	2210419D22Rik	0.0237	-2.020
Hira	0.0424	2.036	Gsdmdc1	0.0205	-2.017
Nup133	0.0151	2.024	St5	0.0280	-2.012
2010107H07Rik	0.0410	2.019	Actb	0.0402	-2.002
9830001H06Rik	0.0329	2.018	Psme1	0.0308	-1.994
2900019M05Rik	0.0063	2.012	LOC674135	0.0102	-1.990
5033430I15Rik	0.0198	2.011	H2-D1	0.0476	-1.989
1110038B12Rik	0.0115	2.007	Trappc6a	0.0206	-1.973
9030013K10Rik	0.0182	2.006	Nagk	0.0341	-1.965
Rassf6	0.0089	2.005	Slc4a3	0.0121	-1.953
Nrn1	0.0236	1.997	Tmem143	0.0289	-1.947
Erlin1	0.0034	1.997	2310005E10Rik	0.0258	-1.944
Lrrc8c	0.0368	1.997	Nov	0.0379	-1.938
Actl6a	0.0467	1.989	Nagk	0.0408	-1.938
Lmcd1	0.0145	1.982	Ctns	0.0366	-1.937
Mrpl9	0.0493	1.981	Naglu	0.0387	-1.931
Hmga1	0.0426	1.978	Emp3	0.0182	-1.927
Smg5	0.0081	1.976	Mrpl23	0.0262	-1.926
Maff	0.0239	1.976	Ddit3	0.0001	-1.925
Ppp1r8	0.0188	1.973	Lamp2	0.0434	-1.917
Stx3	0.0027	1.968	Rasl11a	0.0302	-1.914
Ptp4a2	0.0385	1.967	2310038D14Rik	0.0098	-1.913
Mfsd2	0.0370	1.965	Ift81	0.0258	-1.912

LOC383897	0.0116	1.965	Col20a1	0.0235	-1.902
Odc1	0.0210	1.965	Camta2	0.0095	-1.900
Ang2	0.0245	1.962	6330414G02Rik	0.0124	-1.898
Cacnb2	0.0095	1.957	Cant1	0.0455	-1.896
Ppp1r14b	0.0033	1.956	Stat3	0.0329	-1.893
Ccdc92	0.0428	1.952	Gulp1	0.0264	-1.890
Nat5	0.0029	1.952	Zfp219	0.0232	-1.890
Spast	0.0076	1.940	Gmpr	0.0339	-1.874
Pkp2	0.0071	1.937	Plekha4	0.0209	-1.870
Fam132a	0.0238	1.933	Cyb5	0.0450	-1.867
Gprk6	0.0459	1.932	Dpysl3	0.0186	-1.864
Plac8	0.0254	1.929	H2afy2	0.0496	-1.862
Smpdl3b	0.0055	1.923	Irf3	0.0206	-1.861
Pik3cb	0.0293	1.923	Slc4a3	0.0213	-1.856
Acsbg1	0.0462	1.922	Tm7sf2	0.0497	-1.853
Gprc5a	0.0389	1.922	1700023B02Rik	0.0491	-1.849
2410076I21Rik	0.0390	1.918	Rnf213	0.0451	-1.844
Exosc10	0.0202	1.917	Slit2	0.0173	-1.843
Rassf1	0.0179	1.915	Nrp2	0.0176	-1.833
Ptpn22	0.0396	1.909	1700113I22Rik	0.0288	-1.833
Mrpl9	0.0423	1.908	B230380D07Rik	0.0078	-1.830
E2f4	0.0365	1.908	Fah	0.0252	-1.828
Dut	0.0394	1.908	B3gnt3	0.0123	-1.817
Eif3b	0.0265	1.904	Chmp1b	0.0020	-1.816
Ssx2ip	0.0318	1.904	D11Wsu99e	0.0083	-1.815
Mlp	0.0025	1.903	2810405K02Rik	0.0119	-1.815
Ccnd3	0.0248	1.902	Dbi	0.0008	-1.814
Mybl2	0.0364	1.901	Zfp187	0.0453	-1.812
Jam2	0.0117	1.899	Inpp5k	0.0224	-1.811
Ngfb	0.0185	1.897	Lamp2	0.0365	-1.807
Klhl21	0.0405	1.894	Itgb4	0.0070	-1.796
Ece2	0.0466	1.893	2310005N03Rik	0.0451	-1.794
Ctnnbip1	0.0358	1.889	D11Wsu99e	0.0352	-1.789
Rad23b	0.0256	1.888	D430042O09Rik	0.0469	-1.783

Ppbp	0.0168	1.888	Serpina3g	0.0457	-1.781
Lrp2	0.0434	1.879	Rhbdf1	0.0261	-1.775
1810062G17Rik	0.0001	1.877	Myst4	0.0229	-1.771
Lias	0.0043	1.873	Sdhc	0.0181	-1.768
Melk	0.0405	1.873	Sfrs17b	0.0161	-1.766
Dut	0.0067	1.871	Ptx3	0.0346	-1.766
Ppm1l	0.0130	1.867	BC013529	0.0474	-1.764
Aifm1	0.0070	1.866	Dbi	0.0002	-1.761
EG667190	0.0021	1.864	Adamtsl3	0.0444	-1.759
Dis3	0.0467	1.864	Prickle3	0.0083	-1.759
a2ld1	0.0057	1.864	Sdf2	0.0204	-1.755
Nono	0.0405	1.863	LOC381302	0.0056	-1.755
Hsp90ab1	0.0103	1.859	BC039093	0.0147	-1.754
Pop5	0.0379	1.855	Pdzrn3	0.0326	-1.751
Ppm1l	0.0237	1.855	Dag1	0.0195	-1.751
Foxc2	0.0462	1.851	Rhobtb1	0.0277	-1.751
Pabpc4	0.0345	1.850	Vps53	0.0323	-1.736
Wdr77	0.0197	1.850	Cd109	0.0023	-1.725
Cd44	0.0002	1.849	Tada1l	0.0076	-1.724
Rplp0	0.0042	1.848	Klf7	0.0175	-1.724
EG545056	0.0410	1.846	1190005F20Rik	0.0080	-1.724
Lmcd1	0.0211	1.846	Hint3	0.0119	-1.723
1110008P14Rik	0.0456	1.843	Dcn	0.0063	-1.722
Capzb	0.0286	1.839	Pacs2	0.0433	-1.722
LOC384338	0.0197	1.839	Nme7	0.0140	-1.721
Sh3bgrl2	0.0366	1.838	Pcdha7	0.0216	-1.720
2410004B18Rik	0.0196	1.838	Man2c1	0.0115	-1.719
Ppp1cc	0.0046	1.838	Tgfbrap1	0.0075	-1.717
Mybbp1a	0.0298	1.837	N4wbp5-pending	0.0148	-1.716
Exosc6	0.0443	1.837	Casp8	0.0001	-1.710
Gdnf	0.0177	1.835	Snca	0.0002	-1.709
Dlk2	0.0040	1.835	Zfp579	0.0331	-1.703
Ddx27	0.0451	1.833	Fam171a2	0.0218	-1.702
LOC100046483	0.0191	1.832	Ogt	0.0242	-1.702

Scg5	0.0041	1.829	AI646023	0.0418	-1.702
2810404O06Rik	0.0171	1.827	Prr14	0.0034	-1.702
Camta1	0.0184	1.824	2900024O10Rik	0.0071	-1.700
LOC100047009	0.0163	1.823	Kctd2	0.0371	-1.693
Prpf38a	0.0408	1.819	Lypla1	0.0196	-1.689
Taf5l	0.0024	1.815	Sel1l	0.0314	-1.682
Exoc1	0.0368	1.807	Pon3	0.0314	-1.681
A030007L17Rik	0.0114	1.806	6030429G01Rik	0.0284	-1.678
Tst	0.0138	1.805	5031414D18Rik	0.0321	-1.677
Xpo4	0.0058	1.804	Irf1	0.0220	-1.676
6330505N24Rik	0.0106	1.804	Mif4gd	0.0267	-1.676
2310061F22Rik	0.0100	1.803	LOC100045005	0.0045	-1.675
LOC381578	0.0462	1.796	Fez1	0.0458	-1.674
D730003I15Rik	0.0101	1.792	Igtp	0.0191	-1.672
AU014645	0.0054	1.790	LOC100040592	0.0104	-1.672
LOC215879	0.0312	1.784	P2rx4	0.0432	-1.667
Tsr1	0.0222	1.781	Atp6v1h	0.0254	-1.664
Map3k11	0.0072	1.781	B3gnt1	0.0013	-1.663
LOC624784	0.0465	1.779	Ahnak	0.0052	-1.662
LOC100046298	0.0311	1.773	Crispld2	0.0407	-1.662
Cstf3	0.0430	1.773	1500005K14Rik	0.0205	-1.660
Hsp90ab1	0.0201	1.769	Stx4a	0.0284	-1.658
LOC381898	0.0029	1.768	1110067D22Rik	0.0273	-1.655
Wdr46	0.0204	1.765	Ndfip1	0.0107	-1.653
LOC100047815	0.0138	1.765	Il17rd	0.0450	-1.651
Gnb4	0.0474	1.759	Wisp1	0.0084	-1.650
LOC623453	0.0340	1.759	Odz4	0.0042	-1.646
Ubqln4	0.0142	1.759	Vps25	0.0182	-1.642
2810453I06Rik	0.0006	1.758	Gnpda1	0.0260	-1.634
Smg5	0.0045	1.758	Sfi1	0.0133	-1.631
Mlf1	0.0274	1.758	Rnf113a2	0.0338	-1.631
Aven	0.0308	1.754	Tcf4	0.0229	-1.630
Cd44	0.0488	1.749	Stard8	0.0177	-1.629
2310007G05Rik	0.0264	1.749	Chd3	0.0087	-1.628

Pkn2	0.0441	1.745	Slit2	0.0494	-1.628
Eif4e	0.0080	1.745	Hsd17b4	0.0298	-1.622
Lmcd1	0.0212	1.744	Ppil3	0.0345	-1.617
Glipr2	0.0018	1.743	Etv1	0.0321	-1.607
1110002E23Rik	0.0372	1.742	Aldh3a2	0.0167	-1.606
Denr	0.0059	1.740	Cdk5	0.0366	-1.605
Nef3	0.0134	1.739	Naalad2	0.0137	-1.600
4933424B01Rik	0.0343	1.738	Ctps2	0.0288	-1.600
Eif2s2	0.0169	1.737	Uros	0.0239	-1.600
Med22	0.0008	1.733	Stard8	0.0014	-1.597
Pcsk4	0.0430	1.732	Mitd1	0.0024	-1.594
Polr1d	0.0177	1.732	Vps41	0.0262	-1.592
Urm1	0.0413	1.732	A630076G18Rik	0.0056	-1.588
Plaur	0.0323	1.729	Rab18	0.0222	-1.586
Ikbkap	0.0003	1.727	Ly96	0.0125	-1.583
Nola3	0.0066	1.727	Gnpda1	0.0022	-1.578
Nadk	0.0022	1.727	Gtse1	0.0228	-1.576
Slc20a1	0.0083	1.726	Tctn3	0.0139	-1.576
Nes	0.0363	1.726	LOC100048504	0.0281	-1.575
Mmd	0.0137	1.716	Insig2	0.0438	-1.567
Plekha3	0.0035	1.716	A730085F06Rik	0.0153	-1.565
Pcsk4	0.0223	1.714	Ifitm1	0.0464	-1.565
Siat7b	0.0156	1.713	Klhl13	0.0365	-1.561
LOC632667	0.0077	1.710	Fbln2	0.0188	-1.559
LOC100045796	0.0125	1.710	Eif2ak2	0.0366	-1.558
Atl2	0.0431	1.710	9830134K01Rik	0.0072	-1.557
Gus-s	0.0031	1.709	Pcmtd1	0.0120	-1.557
Pole3	0.0232	1.707	Decr2	0.0406	-1.556
2610019N19Rik	0.0140	1.705	Rragb	0.0126	-1.556
Mlf1	0.0110	1.704	H3f3a	0.0251	-1.556
Sh3bgrl2	0.0169	1.703	2810455F06Rik	0.0255	-1.553
Memo1	0.0353	1.703	Kif3a	0.0079	-1.553
Spc24	0.0226	1.701	Vezf1	0.0114	-1.549
C330023M02Rik	0.0112	1.700	Xpr1	0.0464	-1.547

Zwint	0.0303	1.697	Pde6d	0.0249	-1.546
Schip1	0.0074	1.696	Plekhb2	0.0488	-1.544
LOC100047009	0.0170	1.695	Rdh14	0.0350	-1.540
Gna14	0.0061	1.693	Hint3	0.0129	-1.540
Rbbp4	0.0103	1.690	Gadd45a	0.0034	-1.539
Nup133	0.0455	1.688	Osbpl1a	0.0101	-1.538
Schip1	0.0422	1.687	1810027O10Rik	0.0241	-1.537
Stard7	0.0060	1.687	Vps25	0.0433	-1.536
Sf3b4	0.0032	1.685	Acbd3	0.0018	-1.535
Bcl2l13	0.0125	1.684	2310047M10Rik	0.0323	-1.535
Asxl1	0.0118	1.676	Pnpla8	0.0058	-1.534
Denr	0.0107	1.675	Colec12	0.0004	-1.534
Ddah1	0.0109	1.674	Colec12	0.0015	-1.533
Dgkz	0.0157	1.674	Rerg	0.0258	-1.532
4933424B01Rik	0.0446	1.674	Ptprs	0.0085	-1.532
Wwc1	0.0173	1.673	Stk17b	0.0280	-1.527
2210039O17Rik	0.0160	1.671	LOC100044376	0.0026	-1.526
Mapk14	0.0030	1.669	Rab5b	0.0188	-1.524
Pbp2	0.0311	1.669	LOC100048376	0.0420	-1.524
Nudt21	0.0095	1.669	Rreb1	0.0259	-1.524
1600023H17Rik	0.0305	1.667	Casp8	0.0221	-1.524
Mcrs1	0.0122	1.667	Dnajc18	0.0365	-1.523
Nol6	0.0213	1.665	Pfdn5	0.0433	-1.520
Dusp16	0.0387	1.664	4833414E09Rik	0.0338	-1.520
Ngfb	0.0344	1.660	Sil1	0.0387	-1.518
Cspg4	0.0481	1.660	Ahnak	0.0122	-1.517
Ccl25	0.0235	1.658	Pfdn5	0.0449	-1.516
Cdc42se1	0.0128	1.658	Accs	0.0389	-1.516
Terf2ip	0.0329	1.657	Npc1	0.0431	-1.514
Ppp1r10	0.0376	1.657	Mterfd2	0.0185	-1.513
Nap1l1	0.0094	1.651	Zeb1	0.0230	-1.511
Tpm2	0.0460	1.649	Irf3	0.0202	-1.511
Mycl1	0.0482	1.649	E130207H16Rik	0.0444	-1.511
Khdrbs3	0.0381	1.648	Skz1-pending	0.0324	-1.509

Spast	0.0025	1.647
Usp10	0.0228	1.647
Bcar3	0.0104	1.644
1810047K05Rik	0.0205	1.639
Rbm8a	0.0070	1.639
Snrpb	0.0119	1.638
2510022D24Rik	0.0199	1.638
Spast	0.0127	1.637
Npm3-ps1	0.0213	1.636
LOC380927	0.0198	1.635
2810003C17Rik	0.0024	1.633
Sssca1	0.0402	1.633
Tmem57	0.0434	1.628
Stard7	0.0462	1.627
Wnt5a	0.0417	1.627
Prkx	0.0349	1.627
LOC100047611	0.0002	1.626
Abcf2	0.0453	1.625
Ela1	0.0453	1.624
Eif2s2	0.0030	1.624
Col4a3bp	0.0045	1.621
Stx3	0.0152	1.621
Gpr176	0.0331	1.620
Tjp2	0.0329	1.619
Ythdf2	0.0380	1.619
Ubap2l	0.0027	1.618
Lsg1	0.0348	1.618
Mt2	0.0315	1.616
Jcam2	0.0380	1.612
Med22	0.0162	1.612
Slc15a4	0.0015	1.611
Ddx51	0.0483	1.610
Tomm70a	0.0018	1.608
Poldip3	0.0171	1.605

2610307O08Rik	0.0258	-1.505
Rragb	0.0070	-1.503

Ftsj3	0.0392	1.603
Ciapin1	0.0434	1.603
Mad1l1	0.0116	1.600
Snapc3	0.0256	1.600
Vps72	0.0442	1.599
Hnrnpd	0.0400	1.597
Usp20	0.0122	1.597
BC006662	0.0448	1.596
Ppp2cb	0.0265	1.596
Rai3	0.0273	1.596
A730050C11Rik	0.0023	1.595
Ubap2l	0.0051	1.595
Mrgprf	0.0120	1.594
Mical3	0.0119	1.594
Clcf1	0.0305	1.593
Rasl12	0.0103	1.593
Pgf	0.0407	1.592
D17Wsu92e	0.0282	1.591
Fads3	0.0468	1.590
Cyld	0.0323	1.589
Sh3glb1	0.0169	1.586
Rbm15	0.0338	1.584
LOC381150	0.0326	1.583
Xpo5	0.0038	1.581
LOC668492	0.0083	1.580
Ibrdc3	0.0149	1.580
LOC626152	0.0241	1.579
Wdr32	0.0251	1.578
Dtnb	0.0049	1.576
Ly75	0.0064	1.575
1810009K13Rik	0.0349	1.575
Slc25a25	0.0179	1.573
Nacc1	0.0006	1.571
LOC633945	0.0197	1.571

Rps6ka4	0.0036	1.568
Tmsb10	0.0089	1.567
1110014N23Rik	0.0201	1.566
Tmem50b	0.0108	1.566
G430064E20Rik	0.0064	1.564
LOC278105	0.0297	1.564
LOC235857	0.0090	1.563
Ppp4r1	0.0161	1.563
Ccnd3	0.0151	1.563
Urb2	0.0188	1.562
Rbm16	0.0197	1.561
LOC100047012	0.0050	1.559
H13	0.0440	1.559
Sf3b3	0.0310	1.552
Arl2bp	0.0324	1.551
Ubap2l	0.0499	1.551
Eef1e1	0.0344	1.550
Dusp16	0.0107	1.550
EG433003	0.0404	1.549
Nola2	0.0272	1.549
Mtap	0.0468	1.548
Atic	0.0476	1.548
Wdr82	0.0179	1.544
EG432448	0.0237	1.542
Rplp0	0.0124	1.540
Ran	0.0452	1.539
Phf13	0.0307	1.539
Pla1a	0.0418	1.538
Setd7	0.0083	1.538
Srd5a1	0.0252	1.537
Ubap1	0.0293	1.537
Pusl1	0.0209	1.537
Wdr1	0.0008	1.536
Sfrs6	0.0288	1.531

1110001J03Rik	0.0110	1.531
scl0002507.1_236	0.0315	1.530
Polr2h	0.0176	1.530
Minpp1	0.0008	1.530
Mpv17l	0.0081	1.529
Nudt21	0.0367	1.529
Prpf3	0.0207	1.528
Uba1	0.0472	1.528
1190028F09	0.0442	1.528
Ubap1	0.0373	1.527
Arl2bp	0.0103	1.526
Zcchc17	0.0364	1.522
Rad23b	0.0034	1.522
Lsm1	0.0246	1.521
Ddx31	0.0439	1.521
Loxl4	0.0121	1.520
Mtx1	0.0238	1.520
Prl2c2	0.0364	1.519
Tnc	0.0333	1.516
Pcsk4	0.0261	1.516
Atp5f1	0.0459	1.515
Nckap1	0.0248	1.515
Slc25a44	0.0110	1.515
Slc2a3	0.0057	1.515
Cnpy3	0.0254	1.514
Zw10	0.0461	1.514
Mapk1	0.0395	1.513
4732469G06Rik	0.0470	1.512
Nap1l1	0.0285	1.511
Ube2i	0.0099	1.511
Eif4e3	0.0443	1.511
2810407C02Rik	0.0170	1.509
C230095J06Rik	0.0000	1.509
LOC666025	0.0252	1.508

Zfp607	0.0047	1.508
Rrn3	0.0131	1.508
Rsc1a1	0.0374	1.505
Rad9	0.0263	1.505
Rp23-297j14.5	0.0319	1.504
Tex10	0.0403	1.503
Rnf4	0.0121	1.502
LOC234582	0.0082	1.502
D730042P09Rik	0.0038	1.501
Sprr2k	0.0390	1.501
Pcbp2	0.0252	1.501
